# ‘Citizens’ Attitudes Under Covid19’, a cross-country panel survey of public opinion in 11 advanced democracies

**DOI:** 10.1038/s41597-022-01249-x

**Published:** 2022-03-28

**Authors:** Sylvain Brouard, Martial Foucault, Elie Michel, Michael Becher, Pavlos Vasilopoulos, Pierre-Henri Bono, Nicolas Sormani

**Affiliations:** 1 Sciences Po - CEVIPOF, Paris, France; 2 Institute for Advanced Study in Toulouse & IE University, Segovia, Spain; 3 University of York, York, UK

**Keywords:** Politics, Economics, Society, Government

## Abstract

This article introduces data collected in the Citizens’ Attitudes Under Covid-19 Project (CAUCP), which surveyed public opinion throughout the Covid-19 pandemic in 11 democracies between March and December 2020. In this paper, we present a unique cross-country panel survey of citizens’ attitudes and behaviors during a worldwide unprecedented health, governance, and economic crisis. This dataset investigates the behavioral and attitudinal consequences of multifaceted Covid19 crisis across time and contexts. In this paper, we describe the design of the CAUCP and the descriptive features of the dataset; we also present promising research prospects.

## Abstract (summary)

**Table:** 

Measurement(s)	Public Opinoin
Technology Type(s)	Individual-level survey

## Background & Summary

This article introduces data collected in the Citizens’ Attitudes Under Covid-19 Project (CAUCP), which surveyed public opinion throughout the Covid-19 pandemic from March to December 2020. In this paper, we present the dataset collected in 11 advanced democracies, which is deposited in open access on the *Sciences Po Dataverse*. It constitutes a unique cross-country panel survey of citizens’ attitudes and behaviors during a worldwide unprecedented health, governance, and economic crisis (N = 27,100 unique respondents over four waves).

Since the Covid-19 crisis tapped into all social dimensions, or *crisis characteristics*, it affected most peoples’ lives, and generated unprecedented individual and collective challenges to the public’s health, the economy, and social life^1^. The pandemic has potentially reshaped democratic politics in general and, in particular, has affected attitudes and behaviors on a large range of political issues. We consider that investigating citizens’ adaption and responses to this crisis, as well as their (possibly enduring) consequences is relevant for several reasons. First, according to the *democratic responsiveness* and *accountability* perspectives^2^, governments’ responses to the crisis are conditioned by public opinion. Specifically, in the case of the Covid-19 pandemic, citizens’ (expected) preferences and partisanship have shaped governments’ response to the virus, sometimes at the expense of scientific expertise^3^. Most existing studies indeed link policy responses to the level of political trust, but they generally overlook other dimensions of public opinion–mostly because of a lack of reliable and comparative data^4,5^. Second, citizens’ reactions to Covid-19 related policies are also conditional to political attitudes and emotional reactions to the crisis. Specifically, sociodemographic characteristics, personality traits, ideology, and emotions influence compliance with public health measures^6^. Third, the Covid-19 pandemic has shattered economies and produced a globalized economic crisis of a major magnitude^7,8^. Economic crises, their perception, and the perception of government responsibility normally have significant electoral and attitudinal consequences^9,10^.

The unprecedented crisis triggered by the Covid-19 pandemic has generated an enormous amount of survey research. However, because of limitations in resources, narrow methodologies, or absence of a comparative perspective, few studies allow a comprehensive assessment of public opinion from the outbreak of the crisis in March 2020 onwards. For instance, available cross-country surveys are mostly cross-sectional. Thus, they overlook the evolution of public opinion, and the durability of the effect, throughout a (at least) two year-long eventful crisis^11^. Further, cross-sectional studies cannot establish causal mechanisms on the determinants of public attitude formation and change. On the other hand, existing panel studies are often limited to a single country, falling short of capturing important contextual effects on political behavior^12^ (e.g. in Austria^13^ or the Netherlands^14^). Some studies do combine cross-country comparisons with panel designs through opt-in questions added in already existing panel surveys–although necessarily limiting the breadth of investigation^15^. The CAUCP addresses these issues by providing public opinion panel data from 11 advanced democracies over the year 2020 to examine the longitudinal and cross-country dynamics of citizens’ preferences, perceptions, and behavior.

The CAUCP cross-country panel survey sets to answer four broad research questions, covering four major dimensions of the effects of the Covid19 pandemic on public opinion. First, **[RQ1] Behavioral and attitudinal reaction to Covid-19 related policies** sets to explain the reasons why people are more or less likely to support and comply with public Covid-19 related recommendations and under which circumstances, but also how people acquire information about the pandemic. It addresses the causal mechanisms by which people perceive the salience of the pandemic and their reaction to the discourse of public authorities, media outlets, and other public opinion leaders. Second, **[RQ2] Political Behavior and Attitudes** investigates how people respond to the crisis in terms of political preferences, political trust, and satisfaction, policy evaluation, blame attribution, emotions, and more general preferences around the role of the State. Third, **[RQ3] Electoral Behavior** examines the electoral consequences of the Covid-19 pandemic to evaluate its effects (if any) on prospective electoral behavior (turnout, vote choices and vote intentions), as well as the influence of retrospective vote choice on behaviors and attitudes linked to the Covid-19 crisis. Fourth, **[RQ4] Social Consequences** examines the conditions under which the COVID-19 crisis increases fragmentation within societies and affects social cohesion. The survey examines the dynamics of individual economic well-being, social isolation, occupational trajectories, and patterns of socialization.

## Methods

### Study design

Because of its scale (4 waves, 11 countries) and the constraints of the Covid-19 pandemic, the CAUCP surveys required a resolutely collective research endeavor. The dataset on Citizens’ Attitudes Under Covid19 Project (CAUCP) was developed by Sylvain Brouard and Martial Foucault at the CEVIPOF in Sciences Po (France), in close cooperation with multiple research partners and institutional support. The data collection has been piloted by a steering committee also including Michael Becher (IAST Toulouse and IE University in Madrid) and Pavlos Vasilopoulos (University of York).

### Case selection

The CAUCP study aims at combining a variety of country-level characteristics in order to offer a broad geographical, sanitary, and institutional scope. The project focuses on advanced democracies, it includes respondents of countries from Western Europe (Austria, France, Germany, Italy, Sweden, United Kingdom), Eastern Europe (Poland), the Americas (USA, Brazil) and Oceania (Australia, New Zealand). These countries also vary along several factors pertaining directly to the Covid-19 pandemic: intensity of the pandemic (from hardest hit countries such as Brazil, the USA and the UK, to the countries that have been least affected in terms of health such as Australia or New Zealand), but also type of policy response (from most restrictive countries such as New Zealand; France, and Italy to the less constraining countries such as Sweden, the USA and Brazil). Finally, our case selection includes variation along several institutional dimensions: length of democratic rule, centralized vs. de-centralized/federal governance, and membership to the European Union.

### Survey design

**Fig. 1:**
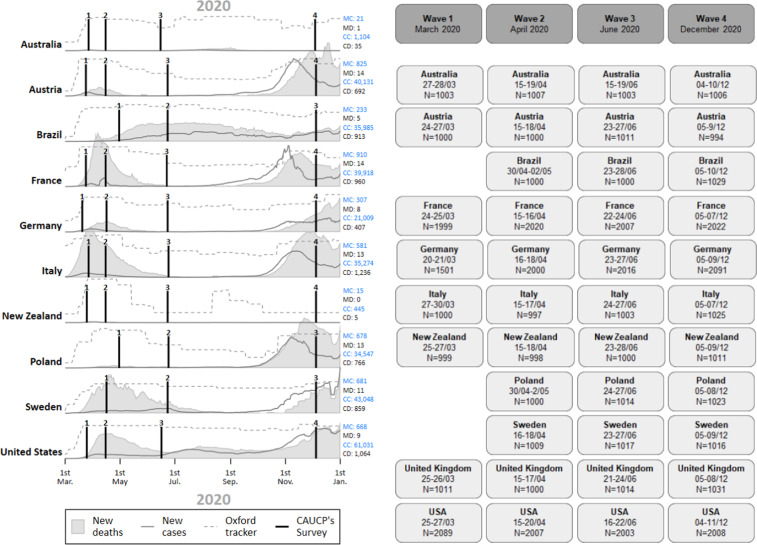
(**a,b**) Design of the cross-country CAUCP panel study.

The CAUCP panel data was collected (Computer-Assisted Web Interview) in four waves, from the outset of the pandemic (Wave 1 in late March 2020) and throughout 2020 (Wave 2 in April 2020, Wave 3 in June 2020, and Wave 4 in December 2020). Therefore, the first wave of the panel covers the implementation of the first health restrictions in mid-March in Europe. The data collection covered the evolution of the pandemic over the summer 2020 (lower intensity of the pandemic and loosened restrictions in the Northern hemisphere vs. higher intensity of pandemic and more stringent policies in the Southern hemisphere), and it extended to December 2020 (as the pandemic dynamic reverses again between South and North). Figure 1a shows the temporality of the waves of the CAUCP panel together with indicators on the strength of the pandemic (number of new cases and covid-related deaths^16^) and on the stringency of governments’ response to the pandemic (Oxford Covid-19 Government Response Tracker^17^). Figure 1b summarizes the fieldwork information (date of data collection and number of respondents for each wave). Because of technical and resource constraints, surveys in Brazil, Poland, and Sweden only started from Wave 2.

### Questionnaire design

The questionnaires of CAUCP surveys was designed on the basis of existing comparative public opinion studies, covering a large array of socio-demographic, political, attitudinal and behavioral characteristics, as well as questions specifically addressing the consequences of the Covid-19 crisis in terms of attitudes, political behavior, support for Covid-19 policies, and the individual socio-economic consequences of the crisis. Questionnaires have been designed and adopted throughout the fieldwork by the CAUCP Steering Committee. The questionnaire retained most questions throughout the waves to allow the longitudinal analysis of variables, but occasionally required some modifications. For instance, questionnaires were flexible between waves and countries in order to include newly salient topics (e.g. compliance with wearing masks from Wave 2 onwards) and to adapt to the countries’ specific contexts (e.g. questions on multi-level governance, or national/local elections). See Table 2 for overview of selected questions included in the questionnaires.

### Ethical validation

The CAUCP data collection has been approved by the Toulouse School of Economics – Institute for Advanced Studies in Toulouse (TSE-IAST) Review Board for ethical Standards in Research in April 2020. The collection of the CAUCP dataset complies with all ethical regulations of public opinion survey data. Participants have been informed of the purpose of the study and consented to sharing their data. Individual data has been entirely anonymized. A similar process is ongoing at Sciences Po to provide the overall ethical approval.

#### Respondents

CAUCP surveyed of total of 27,100 uniquely identified respondents, which constitutes 53,083 observations across the four panel waves. In order to increase the representativeness of the samples, each panel wave includes a minimum of 1,000 respondents in eight countries and about 2,000 in France, Germany, and the USA.

## Data Records

The data generated by the CAUCP project is available in open access on the *Sciences Po Dataverse*^18^. We have uploaded sub-datasets for each wave in each country of the study in TAB format. The country*wave sub-datasets have been cleaned to standardized variable names across country and wave sub-datasets datasets. We have also included detailed codebooks for each for each sub-dataset which describes all the variables included in each sub-dataset (variables labels, answer categories, answer labels, country specific variables) in XLSX format. All this material is available at the following link: https://data.sciencespo.fr/dataset.xhtml?persistentId=doi%3A10.21410%2F7E4%2FEATFBW.

In addition to the individual country*wave files and documentations uploaded on the institutional repository of Sciences Po, we have uploaded a merged dataset on figshare repository^19^. This merged dataset includes the observations for the eleven countries and the 4 waves, as well as an extended codebook in XLSX format, detailing variable types, names, labels, values, value labels, and inclusion or not in each panel wave (this codebook includes generic question wordings, as well as separate tabs for each country which details country-specific questions and answer categories).

## Technical Validation

The CAUCP included a total of 27,100 individual panelists residing in the country where they are surveyed. Online recruitment of the participants began on March 20, 2020 in Germany (Wave 1) and ended on December 11, 2020 in the USA. The CAUCP panel survey lasted for over 10 months between Wave 1 and Wave 4, and the overall full panel retention is low: 3680 respondents participated in all 4 waves (13.6% of the total sample). However, on average, respondents have participated in 1.96 waves – which ensures that almost all respondents were surveyed more than once throughout the pandemic. Table 1 details the panel structure of the CAUCP data, it presents the number of respondents in each wave; the number of respondents from Wave 1 in Waves 2, 3, and 4; and the number of respondents from each preceding wave in Waves 2, 3, and 4.

**Table 1:** Panel structure of CAUCP data.

	Number of Respondents				Respondents from Wave 1			% Panel from Wave 1			Respondents from preceding Wave			% Panel from preceding wave		
	Wave 1	Wave 2	Wave 3	Wave 4	Wave 2	Wave 3	Wave 4	Wave 2	Wave 3	Wave 4	Wave 2	Wave 3	Wave 4	Wave 2	Wave 3	Wave 4
Germany	1501	2000	2016	2091	677	738	728	45,1%	49,2%	48,5%	677	987	1 385	45,1%	49,4%	68,7%
France	1999	2014	2007	2121	1430	1475	1553	71,5%	73,8%	77,7%	1430	1484	1621	71,5%	73,7%	80,8%
United Kingdom	1011	1000	1014	1031	784	726	643	77,5%	71,8%	63,6%	784	746	727	77,5%	74,6%	71,7%
New Zealand	999	998	1000	1011	313	368	300	31,3%	36,8%	30,0%	313	344	342	31,3%	34,5%	34,2%
Austria	1000	1000	1011	994	477	331	396	47,7%	33,1%	39,6%	477	488	494	47,7%	48,8%	48,9%
Italy	1000	997	1003	1025	645	438	609	64,5%	43,8%	60,9%	645	570	688	64,5%	57,2%	68,6%
Sweden	—	1009	1017	1016	—	499	411	—	49,5%	40,7%	—	499	572	—	49,5%	56,2%
Brazil	—	1000	1000	1029	—	462	446	—	46,2%	44,6%	—	462	585	—	46,2%	58,5%
Poland	—	1000	1014	1023	—	424	625	—	42,4%	62,5%	—	424	636	—	42,4%	62,7%
USA	2089	2007	2003	2008	1228	814	640	58,8%	40,6%	31,9%	1228	814	640	58,8%	40,6%	32,0%
Australia	1003	1007	1003	1006	213	509	204	21,2%	50,5%	20,3%	213	182	394	21,2%	18,1%	39,3%

Respondents of the CAUCP panel data were selected through quota sampling, which were based on a combination of multiple socio-demographic characteristics. In the 11 countries of the panel, sampling quotas were based on gender, age, occupation, region of residence, type of environment (town size or population density). In some countries, quota samplings also include additional weighting characteristics, such as ethnicity in the USA, or education in France. The full structure of unweighted samples, weighting coefficients and targeted populations for each country and each wave is presented in Appendix A. In each survey, weighting scores were obtained thanks to RIM weighting (Random Iterative Method–‘Raking’).

Identical questionnaires were administered in all countries for each wave of the CAUCP survey–albeit some questions were adapted to country-specific characteristics (notably with regards to ideology, and political and electoral behavior). On average, respondents took around 15 minutes to answer the questionnaire from Wave 1. Because of the additional questions tailored to the development of the pandemic, questionnaires of the Waves 2, 3 and 4 required more time from respondents, and lasted around 20 minutes. (See Appendix B for average response time of each panel wave in each country).

The CAUCP consists of 317 unique variables measured in all countries (some variables were introduced or drop in different waves). Table 2 presents a selection of the most relevant topics and variables covered in CAUCP data, grouped by main research themes. In addition to the variables presented in Table 2, the CAUCP survey includes extensive information on respondents’ socio-demographic characteristics (gender, age, religiosity, income, education, household description, employment status and trajectory). In this overview, the variables related to a series of randomized experiments carried simultaneously to the CAUCP fieldwork are excluded. Such experiments addressed RQ1 (Behavioral and attitudinal reaction to Covid-19 related policies) and focused on individual compliance to health regulations–data and results are presented separately elsewhere^20^.

**Table 2:** Scope and main variables of interest of the CAUCP survey.

	Electoral Behavior	W1	W2	W3	W4
*Vote*	Prospective Vote	✓	✓	✓	✓
	Retrospective vote choice	✓	✓	✓	✓
	Vote registration	✓	✓	✓	✓
**Political Behavior and Attitudes**					
*Partisanship*	Partisan proximity	✓	✓	✓	✓
	Ideological positioning	✓	✓	✓	✓
*Evaluation*	Perception of the country’s health situation	✓	✓	✓	✓
	Perception of the country’s economic situation	✓	✓	✓	✓
	Change in the country’s health situation in the last 4 weeks			✓	✓
	Change in the country’s economic situation in the last 4 weeks			✓	✓
	Government’s responsibility in health evolution			✓	✓
	Government’s responsibility in economic evolution			✓	✓
	Evaluation economic measures	✓	✓	✓	✓
	Evaluation health measures	✓	✓	✓	✓
	Likelihood patient not treated	✓	✓		✓
*Satisfaction*	Life Satisfaction	✓	✓	✓	✓
	Satisfaction with Head of Government	✓	✓	✓	✓
	Satisfaction with government’s handling of crisis	✓	✓	✓	✓
	Satisfaction with regional government’s handling of crisis	✓	✓	✓	✓
	Satisfaction with democracy		✓	✓	✓
*Trust*	Trust: Big companies		✓		✓
	Trust: Journalists		✓		✓
	Trust: Scientists	✓	✓	✓	✓
	Trust: The government	✓	✓	✓	✓
	Trust: The mayor of your town/city	✓	✓	✓	✓
	Trust: The Prime minister	✓	✓	✓	✓
*Group trust*	Trust: People with a different nationality	✓	✓	✓	✓
	Trust: People with different religious beliefs	✓	✓	✓	✓
	Trust: People you know personally	✓	✓	✓	✓
	Trust: People you’ve met for the first time	✓	✓	✓	✓
	Trust: Your family	✓	✓	✓	✓
	Trust: Your neighbors	✓	✓	✓	✓
*Emotions*	Feeling coronavirus situation: Anger	✓	✓	✓	✓
	Feeling coronavirus situation: Fear	✓	✓	✓	✓
	Feeling coronavirus situation: Hope	✓	✓	✓	✓
*Public Spending*	Public Expenditure: Border control		✓	✓	✓
	Public Expenditure: Business and Industry		✓	✓	✓
	Public Expenditure: Defense		✓	✓	✓
	Public Expenditure: Education		✓	✓	✓
	Public Expenditure: Health		✓	✓	✓
	Public Expenditure: Housing		✓	✓	✓
	Public Expenditure: Pension (Superannuation)		✓	✓	✓
	Public Expenditure: Police and Law Enforcement		✓	✓	✓
	Public Expenditure: Public Transport		✓	✓	✓
	Public Expenditure: The Environment		✓	✓	✓
	Public Expenditure: Unemployment Benefits		✓	✓	✓
	Public Expenditure: Welfare Benefits		✓	✓	✓
**Behavioral and attitudinal reaction to Covid19 policies**					
*Compliance*	Coughing or sneezing into your elbow or a tissue	✓	✓	✓	✓
	Washing your hands more often and/or for a longer amount of time?	✓	✓	✓	✓
	Avoid busy places	✓	✓	✓	✓
	Reduced trips outside	✓	✓	✓	✓
	Stopped greeting by shaking hands, hugging or kissing	✓	✓	✓	✓
	Stopped seeing friends	✓	✓	✓	✓
	Keep a distance of six feet between yourself and other people outside your home	✓	✓	✓	✓
	Leave home less than once a day		✓		✓
	Wear a mask outside your home		✓	✓	✓
	Last 2 weeks: percentage of the population respecting sanitary rules			✓	
*Pandemic Policies*	Aids for firms			✓	✓
	Facilitated access to unemployment insurance for employees			✓	✓
	Financial support for self-employed			✓	✓
	Participation of the State in large firms’ capital			✓	✓
	Rent deferrals for tenants			✓	✓
	Short-term work schemes or furloughing			✓	✓
	General lock-down	✓	✓		✓
	Closing daycares, schools and universities	✓	✓	✓	✓
	Closing non-essential stores	✓	✓	✓	✓
	Health check and mandatory quarantine for people entering the country	✓	✓	✓	✓
	Curfew and using police or the army to control people’s movements	✓	✓	✓	✓
	Mandatory quarantine for all contaminated patients in specific places outside their home	✓	✓	✓	✓
	Mandatory wearing of mask outside home		✓	✓	✓
	Postponing elections	✓	✓	✓	✓
	Prohibiting non-essential trips	✓	✓	✓	✓
	Systematic testing for COVID-19		✓	✓	✓
	Closing borders for foreigners	✓	✓	✓	✓
	Using mobile phone data to control people’s movements	✓	✓	✓	✓
*Information*					
	Likelihood the government is hiding information on coronavirus	✓	✓	✓	✓
	Likelihood scientists are hiding information on coronavirus	✓	✓	✓	✓
	Information about the coronavirus: Printed newspapers				✓
	Information about the coronavirus: Printed newspapers				✓
	Information about the coronavirus: Social media				✓
	Information about the coronavirus: Television				✓
	Information about the coronavirus: Websites or mobile applications other than social media				✓
**Social consequences**					
*Health*	Last few weeks symptoms	✓	✓	✓	✓
	Last few weeks symptoms: family member	✓	✓	✓	✓
	Last few weeks symptoms: friends or acquaintances	✓	✓	✓	✓
	Last few weeks symptoms: household	✓	✓	✓	✓
	Evolution situation last 4 weeks	✓	✓	✓	✓
	Likelihood to be infected if you resume your usual way of life			✓	✓
	Likelihood to be seriously ill if infected by COVID19				✓
	Likelihood to be infected given your current way of life and working conditions	✓	✓	✓	✓
	Accept risk: in general	✓	✓	✓	✓
	Accept risk: in health matters	✓	✓	✓	✓
	Accept risk: in making political choices	✓	✓	✓	✓
	Conditions: Cardiovascular diseases, diabetes, hepatitis B, chronic obstructive pulmonary disease, chronic kidney diseases, and cancer.	✓	✓	✓	✓
	Positive COVID19 since the start of 2020?				✓
	Health country consequences	✓	✓	✓	✓
	Agree to be vaccinated				✓
	Income evolution between November and January 2020		✓	✓	✓
*Economy (egotropic)*	Expected household pre-tax income to decrease in 2021 compared to 2020				✓
	Occupation as of January 1st, 2020		✓	✓	✓
	Occupation today		✓	✓	✓
	Likelihood to be unemployed by March 31, 2021				✓
	Last two weeks: Feeling down, depressed, or hopeless	✓	✓	✓	✓
*Social isolation*	Last two weeks: Little interest or pleasure in doing things	✓	✓	✓	✓
	Last two weeks: heard from friends		✓		✓
	Last two weeks: heard from relatives		✓		✓
	Close friend to call help		✓		✓
	Relatives to call for help		✓		✓
	Last two weeks: Felt isolated from others		✓		✓
	Last two weeks: Felt left out?		✓		✓
	Last two weeks: Felt that you lack companionship?		✓		✓

## Usage Notes

To conclude the presentation of the CAUCP dataset, we present three key dimensions covered by our survey: *policy compliance*, *attitudinal dynamics*, and *democratic accountability*. We describe three key variables to study the possibly large and lasting effects of the Covid-19 crisis on social and political life.

### Public health policy compliance

**Fig. 2:**
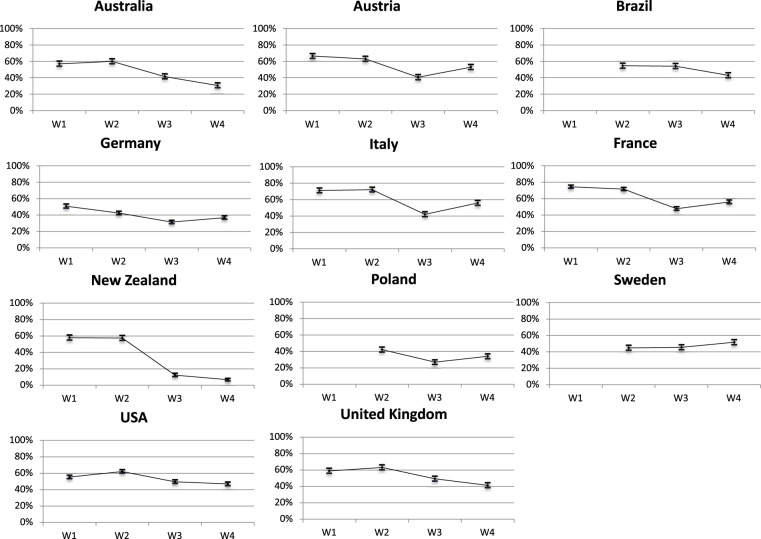
Compliance: Keeping distance with people outside home. Question wording: “Due to the coronavirus epidemic, in your daily life, would you say that you keep a distance of three feet (1 meter) between yourself and other people outside your home? 0 means “not at all” and 10 means “yes completely”. shows the proportion of respondents who score 10. We consider that the latter respondents are those expressing full compliance to restrictive measures.

Compliance with Covid-19 related policies is both a salient political debate and constitutes a promising research agenda. Indeed, the preventive and sometimes restrictive policies implemented during the Covid-19 pandemic have been extensively discussed in the public debate. We identify two dimensions of *policy compliance*: individual behavior (i.e. respecting restrictive regulations) and acceptance/evaluation of Covid-19 related measures (i.e. support for policies restricting individual and civil liberties). Despite sharp political volatility, such as decline (or variations) in trust and negative evaluations of the government, compliance levels to stringent policies remained high^21,22^ (Newton 2020). However, compliance is mediated by partisanship: support for the parties in government leads to greater compliance^23,24^. The politics of complying to stringent health policies is certainly influenced by individual and contextual factors. For instance, patterns of compliance to ‘keeping social distance’ diverge across countries, but also evolve over time (Fig. 2). Even in the case of New Zealand which has been less exposed to the coronavirus in terms of fatalities, citizens do react to the Covid-19 related policies and adapt to government recommendations. Indeed, pandemic policies are the most visible policies: virtually all citizens are aware and informed on Covid-19 restrictions. This constitutes a unique research area to evaluate both individual and contextual determinants of policy acceptance.

### Attitudinal dynamics

**Fig. 3:**
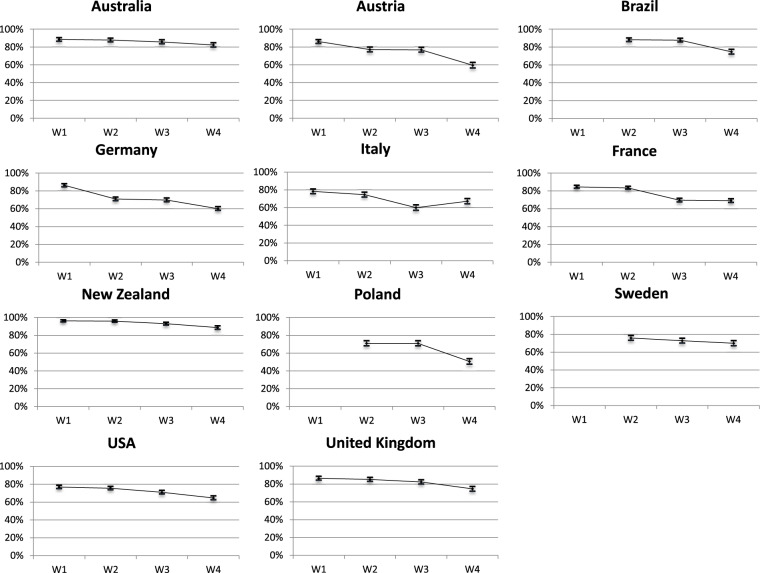
Preferences for closing the borders to foreigners. Question wording: “Here is a list of measures that have been taken in some countries against the spread of coronavirus (N-Covid19). Do you agree with them? The closing of the borders for foreigners” - 5-point Likert Scale. shows the proportion of respondents who “completely agree” or “tend to agree”.

The Covid-19 pandemic had broad consequences for public attitudes. Many pundits and commentators speculate that the Covid-19 may deeply modify political cleavages. The pandemic may also polarize or de-polarize democratic societies. Alternatively, the political impact of the pandemic may be sectoral only, that is, limited to the specific dimensions linked to the crisis. Finally, the Covid-19 pandemic might not be an influential crisis, as long as citizens’ attitudes are concerned, compared to previous major crises, such as geopolitical shocks or economic crises. Still, findings suggest that the pandemic has triggered important attitudinal changes, which however mask individual-level heterogeneity within each country of our sample (for instance Galasso *et al*. find sizeable gender differences in attitude during the crisis^25^). What are the factors driving these attitudinal changes? As new issues appeared on the public agendas, there are also opportunities to study the formation of attitudes. Furthermore, many psychological traits, such as personality characteristics, risk perception, emotions are expected to influence political attitudes. Even more, the enormous amount of (mis-) information on the Covid-19 pandemic may have also influenced political attitudes. Indeed, earlier findings have found evidence that different patterns of news consumption affected levels of trust and compliance with Covid-19 related restrictions^22^. Descriptive findings of the CAUCP survey tend to indicate that the pandemic has not transformed overall political preferences. Indeed, average ideological preferences (Left-Right 11-point scale) are stable over the waves of the panel, and polarization seems unchanged (roughly measured with the standard deviation of the Left-Right scale). Despite a political anchor such as ideology remaining stable throughout the crisis other attitudes tend to fluctuate across time and contexts. For instance, preferences for closing borders have changed during the crisis, although in contrasted directions (Fig. 3).

Even further, the political consequences of the pandemic seem to be mediated by anxiety rather than a cognitive trigger of the political ‘rally’ effect^26^. The CAUCP offers a promising agenda for research in political psychology with a large battery of questions on emotions and social interactions.

### Democratic accountability

**Fig. 4:**
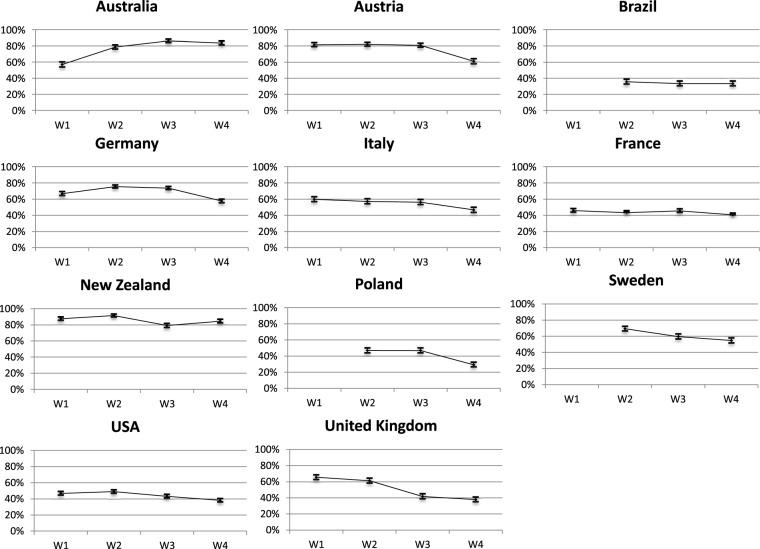
Satisfaction with the government’s handling of the Covid19 crisis. Question wording: “Generally speaking, are you satisfied with the way that the government is handling coronavirus?” – 4-point Likert Scale. shows the proportion of respondents that are “completely satisfied” or “quite satisfied”.

Studying the consequences of the Covid19 pandemic in terms of democratic accountability represents a rich research agenda. Citizens’ responses to the unprecedented pandemic policies, and to the subsequent expected economic crisis, is crucial to understand the functioning of democracies and will most likely be multifaceted (electoral behavior, trust and evaluation of institutions and policies, blame attribution). For instance, initial findings on citizens’ response to the Covid-19 pandemic show a ‘rally around the flag effect’^27,28^. However, this effect should be qualified since it is neither universal^29^ nor similarly lasting as the crisis endures^30^, or rather producing a ‘*crisis signal effect that benefits incumbents*’^31^. Research suggests that the pandemic has triggered stronger support or satisfaction with the governments and their policy – largely mediated by levels of trust and partisanship^32^. The CAUCP data confirms that citizens’ policy response is complex and context dependent. Looking at the satisfaction with the government’s handling of the Covid19 crisis, we observe important time and country variation (Fig. 4). Indeed, in the different countries, the level of satisfaction is extremely high (Australia, New Zealand, Austria), average (France, Italy, USA), or generally low (Brazil, Poland). Additionally, trends of satisfaction are either increasing (Australia), stable (France, New Zealand), or declining (Sweden, United Kingdom). In addition to attitudes toward the government, social science research should further investigate the electoral consequences of the crisis. Indeed, local sanitary contexts and corresponding health policies have affected turnout rates in several European local elections, increasing turnout in some cases^33^ or depressing it in others^34,35^. Further, the Covid19 pandemic has influenced voting behavior, generally to the benefit of incumbents and Green parties in local elections^36^. How consistent are these patterns? Are they dependent on the context of the pandemic and the policy response? Do they hold for national and local elections? Such questions enable to investigate promising research venues on the effects of retrospective vote choice on attitudes, as well as on the consequences of the crisis on prospective vote choice, and actual electoral behavior as three countries held national elections in 2020 (USA, New Zealand, and Poland). Evaluating the significance of individual-level health and economic situations on political behavior and attitudes constitute a promising research lead, as most studies examined these questions at the aggregate level thus far.

## Supplementary information

Appendix - A

Appendix B

## Data Availability

The country*waves files of the CAUCP dataset have been merged using STATA16. The Code is available on the figshare repository.
